# Process Optimization and Quality Characterization of *Indocalamus latifolius* Leaf–White Tea

**DOI:** 10.3390/foods15101676

**Published:** 2026-05-11

**Authors:** Chengjie Wang, Chan Huang, Yujue Zhao, Xinyu He, Xi Cheng, Jianming Zhang, Haoran Chen, Jianfeng Huang, Yan Huang, Shaoxiao Zeng

**Affiliations:** 1 College of Food Science, Fujian Agriculture and Forestry University, Fuzhou 350002, China; 14736814690@163.com (C.W.); 17807851896@163.com (C.H.); 18176289810@163.com (Y.Z.); chenhaoran6054@163.com (H.C.); hjfeng2023@163.com (J.H.); 2 College of Tea and Food Science, Wuyi University, Wuyishan 354300, China; 15819914459@163.com (X.H.); wyxychengxi@wuyiu.edu.cn (X.C.); zjm0308@163.com (J.Z.); 3 Fujian Provincial Key Laboratory of Quality Science and Processing Technology in Special Starch, Fujian Agriculture and Forestry University, Fuzhou 350002, China

**Keywords:** *Indocalamus latifolius* leaf–white tea, process optimization, chemical composition, metabolomics

## Abstract

White tea (WT) and *Indocalamus latifolius* leaves (ILLs) are valued for their distinctive aromas and bioactive properties. In this study, flavonoid extraction from ILLs was optimized using total flavonoid yield as the response variable through single-factor experiments and response surface methodology. The resulting *Indocalamus latifolius* leaf extract (ILLE) was vacuum-concentrated and sprayed onto WT to produce *Indocalamus latifolius* leaf–white tea (IT). The effects of spray ratio, spraying liquid temperature, and drying temperature on the sensory and chemical qualities of IT were further evaluated. Untargeted metabolomics was performed to compare the metabolite profiles of IT and WT. The optimal extraction conditions for ILLs’ flavonoids were 95 °C, 120 min, and a liquid-to-solid ratio of 60:1 mL/g, yielding 4.39 mg/g total flavonoids. The optimal processing parameters for IT were a spray ratio of 1:1.75 g/g, a spraying liquid temperature of 50 °C, and a drying temperature of 105 °C. Compared with WT, IT showed improved sensory and biochemical qualities, including a richer and more persistent ILLs aroma, a more refreshing and more layered taste, and significantly higher levels of water extracts, flavonoids, tea polyphenols, soluble sugars, and amino acids (*p* < 0.05). Metabolomic analysis revealed clear compositional differentiation between IT and WT, including the enrichment of several metabolites, some with potential functional relevance, as well as aroma-active compounds. These findings provide chemical and sensory evidence for the distinctive quality of IT and support further development of this novel specialty tea.

## 1. Introduction

Tea, originating in China, is the world’s second-most-consumed natural beverage after water. It is classified into six major types based on its processing methods: green tea, white tea, oolong tea, black tea, yellow tea, and dark tea [1]. White tea (WT) is a lightly fermented tea primarily produced in the eastern and northern regions of Fujian Province, particularly in Fuding City and Zhenghe County, which are known as the core production areas [2]. Due to its delicate floral fragrance, refreshing taste, and pharmacological properties such as antioxidant, anti-inflammatory, and metabolic regulatory effects, WT has gained increasing global attention and market acceptance in recent years [3,4]. Currently, the mainstream WT products are traditional varieties like Baihao Yinzhen (Silver Needle) and Bai Mudan (White Peony), which remain focused on conventional sensory styles. These products may not fully meet the increasing consumer demand for more diverse flavor experiences and differentiated product characteristics. In this context, reprocessed tea, as an innovative processing strategy, introduces exogenous plant extracts with synergistic effects. By retaining the inherent qualities of WT, this approach creates composite WT products with novel flavor characteristics and enhanced functional properties, thereby effectively improving product differentiation and adding value to the industry.

Currently, reprocessed tea has evolved into a diversified product system, with flower teas accounting for the largest share and the most mature industrialization. Typical examples include jasmine tea, osmanthus tea, rose tea, and gardenia tea, all of which have achieved large-scale production and widespread market recognition [1,5]. The key to flower tea processing lies in the “scenting” process, which is primarily divided into traditional scenting and isolated scenting [6]. In traditional scenting, tea leaves are directly mixed with fresh flowers. After absorbing the fragrance, the flowers are removed, and the tea is dried to stabilize the quality. In isolated scenting, the tea and fresh flowers are separated by a physical barrier, such as a mesh or breathable membrane, allowing the fragrance to transfer through the movement of air. Despite the increasing variety of flower teas, their processing largely focuses on the passive transfer of volatile aromatic compounds, with limited ability to extract and integrate non-volatile active functional ingredients, such as flavonoids and polyphenols, from the flowers. Furthermore, functional composite teas developed with medicinal and edible plants (e.g., ILLs, mulberry leaves, etc.) as supplementary ingredients have yet to establish systematic research or industrial applications at both the product structure and technological pathway levels. Therefore, breaking through the limitations of traditional scenting techniques and constructing a novel composite tea processing system that integrates both fragrance enrichment and synergistic enhancement of active ingredients is of great scientific significance and practical value. This approach may expand the flavor spectrum of tea products and promote innovation in the tea industry.

*Indocalamus latifolius* leaves (ILLs) are native to China and possess a delicate, long-lasting natural fragrance [7]. The leaves are broad, flexible, and highly resistant to steaming, making them a traditional material for wrapping zongzi, a traditional food during the Dragon Boat Festival. Modern pharmacological research has confirmed that ILLs are rich in flavonoids, polyphenols, and various volatile aromatic compounds, which exhibit significant antioxidant and antimicrobial activities, as well as medicinal properties such as heat-clearing, dampness-reducing, and thirst-quenching effects [8,9]. However, the current application of ILLs is limited, primarily used as a single-use wrapping material that is often discarded or burned after use. This results in inefficient utilization of renewable plant resources and contributes to environmental pollution due to smoke emissions from burning. Therefore, systematically exploring the extraction and high-value utilization of ILLs’ active compounds is of significant scientific importance and practical value, promoting the resource-efficient, environmentally friendly, and industrial development of agricultural by-products.

In this study, *Indocalamus latifolius* leaf extract (ILLE) was combined with WT through a spray-assisted adsorption approach. Unlike conventional scenting processes that rely exclusively on the passive transfer of volatile aroma compounds, this method facilitates the efficient penetration and enrichment of polyphenols, flavonoids, and characteristic aromatic compounds from ILLs into the WT matrix, representing a fundamental innovation in both processing principle and product composition. Consequently, the resulting IT exhibits a distinctive flavor quality and an altered compositional profile. This study provides a new pathway for the efficient utilization of ILLs and offers theoretical support and practical insights for the development of new tea products.

## 2. Materials and Methods

### 2.1. Chemicals and Materials

WT and ILLs were supplied by Zhenghe Yungen Tea Co., Ltd. (Nanping, China). Anhydrous ethanol was obtained from China National Pharmaceutical Group Chemical Reagent Co., Ltd. (Shanghai, China). Rutin, acetonitrile, sodium hydroxide, sodium nitrite, 2-chloro-L-phenylalanine, octyl alcohol, and formic acid were procured from Aladdin Bio-Chem Technology Co., Ltd. (Shanghai, China). Methanol and acetonitrile were sourced from Thermo Fisher Scientific (Waltham, MA, USA). Formic acid was acquired from TCI (Tokyo, Japan), ammonium formate and n-alkanes from Sigma-Aldrich (St. Louis, MO, USA), and n-hexane from Yonghua Chemical Technology Co., Ltd. (Changshui, China).

### 2.2. ILLE Preparation Process Experiment

#### 2.2.1. Single-Factor Experiment

Distilled water was used as the extraction solvent for all ILLE preparation experiments, and the solvent system was kept consistent throughout the extraction optimization process. Total flavonoid yield was selected as the response variable because flavonoids are among the representative functional constituents reported in *Indocalamus latifolius* leaves, and preliminary experiments indicated that flavonoid yield was sensitive to changes in extraction conditions, making it a practical indicator of extraction efficiency. In a single-factor experimental design, the total flavonoid extraction yield was used as the response variable, and the effects of three factors on the extraction rate of total flavonoids from ILLs were investigated: extraction temperature (60, 70, 80, 90, and 100 °C), extraction time (60, 90, 120, 150, and 180 min), and liquid-to-solid ratio (30:1, 45:1, 60:1, 75:1, and 90:1 mL/g). No additional pH adjustment was applied during extraction, and the process was conducted under the natural pH of the solvent system.

#### 2.2.2. Response Surface Methodology (RSM) Experimental Design

On the basis of the single-factor results, RSM was applied to further optimize the extraction of total flavonoids from ILLs. Extraction temperature, extraction time, and liquid-to-solid ratio were used as independent variables, while total flavonoid yield served as the response. A second-order polynomial regression model was constructed, and the model’s goodness of fit and factor interactions were evaluated using analysis of variance (ANOVA) and significance testing of the regression coefficients (*p* < 0.05). The levels of these factors are shown in Table 1.

#### 2.2.3. IT Processing Technology

The preparation of IT involves three key steps: ILLE preparation, WT infusion and adsorption, and drying. Specifically, ILLE was prepared based on the optimal extraction process identified in Section 2.2.2. The extract was concentrated using a rotary evaporator (Shanghai Yarong Biochemical Instrument Factory, Shanghai, China) at 60 °C under reduced pressure until the final volume reached one-eighth of the original extract volume, yielding the concentrated ILLE used for subsequent spraying experiments. After preheating the concentrated liquid to the desired temperature, it was sprayed onto the surface of WT leaves using a manual spray bottle (nozzle size: 1 mm) according to the designated spraying ratio. The tea leaves were continuously mixed during spraying until they reached saturation and were then dried to a moisture content of ≤6.5%. To optimize the process, single-factor experiments were conducted to investigate the effects of spraying ratio (1:1, 1:1.25, 1:1.5, 1:1.75, and 1:2 g/g; expressed as the mass ratio of concentrated ILLE to dry WT), spraying temperature (20, 30, 40, 50, and 60 °C), and drying temperature (60, 75, 90, 105, and 120 °C) on the total flavonoid content in IT. This stepwise spraying-ratio gradient was designed to evaluate the adsorption–saturation behavior of the WT matrix and the flavonoid-enrichment efficiency under increasing ILLE loading, thereby identifying a processing level that balances enrichment efficiency, avoidance of excessive residual liquid, and practical operability.

### 2.3. Sensory Evaluation Procedure

Sensory evaluation was conducted by six trained panelists (three males and three females, aged 30–50 years) from Fujian, China, each with more than 10 years of experience in white tea evaluation. Since this study aimed to perform a comparative expert assessment of processing effects rather than a consumer acceptance test, a small trained panel was considered appropriate for obtaining consistent and professional sensory judgments. The method was adapted from Huang et al. [10]. Briefly, 3.0 g of the tea sample was infused in boiling water at a 1:50 (*w*/*v*) ratio for 5 min, after which the infusion was filtered. Appearance, liquor color, aroma, and taste were scored independently on a 0–100 scale, and the final score for each attribute was calculated as the average of the panelists’ scores.

### 2.4. Determination of Biochemical Components 

Water extracts’ content in tea samples was determined following the method of Li et al. [11]. Total polyphenols, total flavonoids, amino acids, and soluble sugars were determined using the Folin–phenol colorimetric method (absorbance at 765 nm, with gallic acid as the standard), the aluminum trichloride colorimetric method (absorbance at 415 nm, with rutin as the standard), the ninhydrin colorimetric method (absorbance at 570 nm, with L-theanine as the standard), and the anthrone–sulfuric acid colorimetric method (absorbance at 620 nm, with glucose as the standard), respectively. The corresponding calibration curves are provided in the Supplementary Materials, Figures S1 and S2.

### 2.5. Liquid Chromatography–Mass Spectrometry (LC-MS) Analysis of Non-Volatile Compounds

#### 2.5.1. Sample Preparation for LC-MS Analysis

Tea samples were first ground and sieved through a 100-mesh screen. A measured amount of the resulting powder was transferred into a 2 mL centrifuge tube, followed by the addition of 600 µL of methanol solution containing 2-chloro-L-phenylalanine. After vortexing for 30 s, 100 mg of glass beads were added, and the mixture was homogenized at 60 Hz for 90 s. The sample was then sonicated at room temperature for 15 min and centrifuged at 12,000 rpm at 4 °C for 10 min. The supernatant was passed through a 0.22 µm membrane filter and placed in a sample vial for LC-MS analysis.

#### 2.5.2. LC-MS Analysis

Non-targeted metabolomic profiling was carried out using a Thermo Vanquish ultra-high-performance liquid chromatography system coupled with a Thermo Q Exactive Focus mass spectrometer (Thermo Fisher Scientific, USA). Separation was achieved on an ACQUITY UPLC^®^ HSS T3 column (2.1 mm × 150 mm, 1.8 µm; Waters Corporation, Milford, MA, USA) at a flow rate of 0.25 mL/min, a column temperature of 40 °C, and an injection volume of 2 µL. The mobile phases were 0.1% formic acid in acetonitrile and 0.1% formic acid in water. MS data were acquired in both positive and negative ionization modes. The spray voltages were 3.50 kV and −2.50 kV for positive and negative modes, respectively, with sheath gas and auxiliary gas flow rates of 30 and 10 arb. The capillary temperature was set at 325 °C.

### 2.6. Gas Chromatography–Mass Spectrometry (GC-MS) Analysis of Volatile Compounds

#### 2.6.1. Sample Preparation for GC-MS Analysis

For volatile analysis, 0.5 g of the tea sample was placed in a 20 mL headspace vial and mixed with 10 µL of internal standard solution, followed by incubation at 60 °C for 10 min. The SPME fiber was preconditioned at 270 °C for 10 min before extraction. It was then exposed to the sample headspace at 60 °C for 15 min and immediately transferred to the GC injector for desorption at 250 °C for 5 min. After each analysis, the fiber was reconditioned at 270 °C for 10 min. For retention-index calibration, 10 µL of n-alkanes was added to a separate headspace vial and analyzed using the same extraction and injection procedure.

#### 2.6.2. GC-MS Detection

Volatile compounds were analyzed using an Agilent 8890A gas chromatograph (Agilent Technologies, Santa Clara, CA, USA) coupled to a LECO Pegasus BT 4D mass spectrometer (LECO Corporation, St. Joseph, MI, USA). Separation was performed on a DB-HeavyWax column (30 m × 250 µm × 0.5 µm; Agilent Technologies, Santa Clara, CA, USA) with high-purity helium as the carrier gas at 1.0 mL/min. The oven program started at 50 °C for 2 min, then increased at 5 °C/min to 240 °C and was held for 5 min. The transfer line and ion source temperatures were both maintained at 250 °C. Spectra were collected at 10 spectra/s under electron-impact ionization at 70 eV with a detector voltage of 1960 V, and the scan range was *m/z* 35–550.

### 2.7. Statistical Analysis

Graphs were generated using Origin 2024 (OriginLab Corporation, Northampton, MA, USA) and GraphPad Prism 8.0 (GraphPad Software, San Diego, CA, USA). Exact *p*-values and detailed statistical outputs for key comparisons are provided in Supplementary Tables S1 and S2.

## 3. Results and Discussion

### 3.1. Single-Factor Experiments for ILLE Preparation

#### 3.1.1. Effect of Extraction Temperature on Flavonoid Yield from ILLs

**Figure 1:**
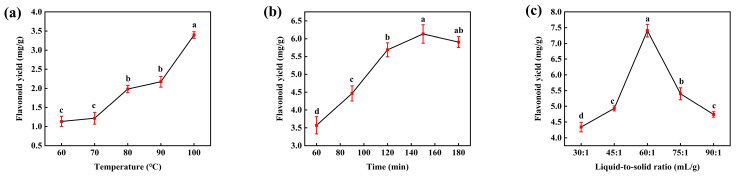
Effects of single factors on flavonoid yield: (**a**) extraction temperature, (**b**) extraction time, and (**c**) liquid-to-solid ratio. Data are presented as mean ± SD. Different lowercase letters indicate statistically significant differences among groups at *p* < 0.05.

Extraction temperature is a critical determinant of the extraction efficiency of flavonoid compounds from plant matrices. Numerous studies have demonstrated a positive association between extraction temperature and flavonoid yield [12,13]. As shown in Figure 1a, within the range of 60–100 °C, the flavonoid yield increased significantly with increasing temperature (*p* < 0.05). This trend can be attributed to enhanced molecular thermal motion at elevated temperatures, which reduces solvent viscosity and interfacial tension, thereby improving solute solubility and diffusion kinetics and ultimately promoting flavonoid extraction [14].

#### 3.1.2. Effect of Extraction Time on Flavonoid Yield from ILLs

Extraction time is another key parameter governing the yield of flavonoids from plant materials. Previous studies have shown that insufficient extraction time can result in incomplete solubilization of flavonoids, whereas overly prolonged extraction may promote thermal degradation and/or oxidative decomposition, thereby markedly reducing the total flavonoid yield. Therefore, systematic optimization of this parameter is of substantial practical importance [15,16]. In the present study, within the range of 60–150 min, the flavonoid yield increased significantly with increasing extraction time. However, when the extraction time was extended to 180 min, the yield began to decline (Figure 1b). These results indicate that moderate prolongation of extraction time facilitates flavonoid release, while beyond a critical threshold, degradation processes become dominant, leading to reduced recovery [17]. This pattern is consistent with observations reported by Wu, Zhao, and colleagues in their investigation of flavonoid extraction from black jujube fruits [14].

#### 3.1.3. Effect of Liquid-to-Solid Ratio on Flavonoid Yield from ILLs

The liquid-to-solid ratio is a key process parameter affecting the extraction yield of flavonoids from plant materials. As shown in Figure 1c, increasing the liquid-to-solid ratio from 30:1 to 60:1 mL/g significantly enhanced flavonoid yield. However, further increases in the liquid-to-solid ratio led to a marked decline in yield. This non-monotonic trend likely reflects the combined effects of mass-transfer kinetics and solvent dilution. At relatively low liquid-to-solid ratios (≤60:1 mL/g), increasing solvent volume decreases the local solute concentration, alleviates mass-transfer resistance at the solid–liquid interface, and enlarges the effective extraction area, thereby facilitating the dissolution and diffusion of flavonoids from the plant matrix [14]. In contrast, at higher liquid-to-solid ratios (≥60:1 mL/g), excessive solvent can over-dilute the target compounds and promote the co-extraction of non-target constituents (e.g., polysaccharides and phenolic acids). These co-extracted components may interfere with quantification or effectively dilute the target fraction and may also hinder sustained flavonoid release via competitive solvation and/or micellar encapsulation effects. Collectively, these factors can manifest as an apparent reduction in the measured total flavonoid yield [18].

### 3.2. Response Surface Optimization for ILLE Preparation

#### 3.2.1. Experimental Design and Results of the RSM

Based on the optimal ranges identified in the single-factor experiments, a Box–Behnken design was employed to perform RSM optimization. Extraction temperature (A), extraction time (B), and liquid-to-solid ratio (C) were selected as independent variables, while total flavonoid yield was defined as the response variable (Table 2). The experimental data were fitted using multivariate regression analysis, yielding the following second-order polynomial model: Y = 4.288 + 0.75375 A + 0.468 B + 0.06025 C − 0.0255 AB + 0.064 AC − 0.1165 BC − 0.8165 A^2^ − 0.217 B^2^ − 0.3375 C^2^.

A one-way analysis of variance (ANOVA) was performed to evaluate the response surface model, and the results are summarized in Table 3. The quadratic model was statistically significant overall (*p* < 0.01; F = 6.95). The coefficient of determination (R^2^) was 0.8993, and the adjusted R^2^ was 0.7699, indicating moderate model performance within the tested experimental range. Examination of individual terms showed that the linear effects of A and B and the quadratic term A^2^ significantly influenced total flavonoid yield (*p* < 0.05), whereas factor C and several interaction terms were not significant (*p* > 0.05). In the present study, the complete quadratic model was retained to preserve the standard structure of the response surface model and to support process optimization within the investigated range. However, because the explanatory ability of the model was moderate and the lack-of-fit *p*-value was close to the significance threshold (*p* = 0.0652), the model results should be interpreted with appropriate caution. Accordingly, this model is better regarded as a practical optimization tool within the studied domain rather than a high-precision predictive model.

#### 3.2.2. Interaction Effect Analysis

**Figure 2:**
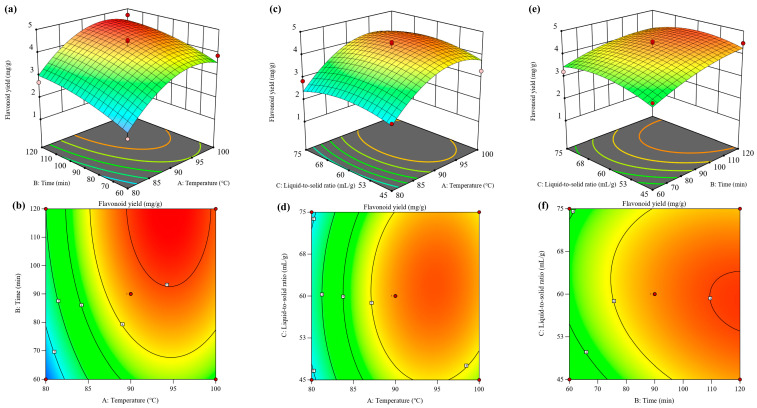
Response surfaces for flavonoid extraction yield. Effects of the interactions between extraction temperature and extraction time (**a**,**b**), extraction temperature and liquid-to-solid ratio (**c**,**d**), and extraction time and liquid-to-solid ratio (**e**,**f**) on the total flavonoid yield.

The three-dimensional response surface plots and their corresponding contour maps generated using Design-Expert 13.0 provide an intuitive visualization of how interactions between extraction factors influence the total flavonoid yield of ILLs. In general, a steeper response surface or markedly elliptical/irregular contour patterns indicate stronger interactions between two variables, whereas a relatively flat surface and near-circular contours suggest weak interaction effects [14]. As shown in Figure 2, the response surface associated with extraction temperature (A) exhibited the steepest slope, followed by extraction time (B), while the surface for the liquid-to-solid ratio (C) was comparatively flat. Accordingly, the relative magnitude of the main effects on total flavonoid yield can be inferred as A > B > C. Moreover, consistent with the contour plot patterns and the ANOVA results (all pairwise interaction terms, *p* > 0.05), no statistically significant interactions were observed between any two of the factors A, B, and C.

#### 3.2.3. Optimization and Validation

The response surface model predicted the optimal extraction conditions as follows: an extraction temperature of 95 °C, an extraction time of 120 min, and a liquid-to-solid ratio of 60:1 mL/g. Under these conditions, three parallel validation experiments yielded an average total flavonoid content of 4.39 mg/g, which was close to the model-predicted value, indicating that the optimized conditions were feasible and acceptable for process optimization within the tested range. Nevertheless, given the moderate adjusted R^2^ and the borderline lack-of-fit result, the model should be interpreted as providing practical guidance for optimization rather than strong predictive power beyond the present experimental domain.

### 3.3. Results of IT Processing Trials

#### 3.3.1. Effect of Spraying Ratio on the Adsorption Performance of IT

The effects of different spraying ratios on WT adsorption are summarized in Table 4. As the spraying ratio increased, the adsorption capacity, adsorption rate, and time to reach saturation all initially increased and then gradually plateaued. This trend can be explained by the increasing availability of adsorption sites on the WT matrix as the spraying ratio increased. At lower spraying ratios, there were still many available adsorption sites on the surface and within the pores of the WT, allowing for further adsorption as more spraying liquid was applied. This resulted in a continued increase in the adsorption capacity. However, when the spraying ratio reached 1:1.75 (g/g), the adsorption capacity reached 5.29 ± 0.10 g, and the adsorption rate was 105.8 ± 2.0%. No statistically significant difference was observed between this group and the 1:2 (g/g) group (*p* > 0.05), but both were significantly higher than the other experimental groups (*p* < 0.05). These findings indicate that at a spraying ratio of 1:1.75 (g/g), the WT matrix approached its adsorption capacity limit, and as the balance of wettability, penetration, and attachment was reached, the adsorption rate began to plateau.

#### 3.3.2. Effect of Spraying Ratio on Flavonoid Yield in IT

**Figure 3:**
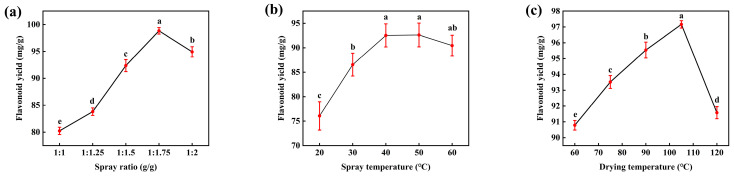
Single-factor experimental analysis of flavonoid yield: (**a**) spraying ratio, (**b**) spraying temperature, and (**c**) drying temperature. Data are presented as mean ± SD. Different lowercase letters indicate statistically significant differences among groups at *p* < 0.05.

As the spraying ratio increased, the total flavonoid yield in IT initially increased and then gradually plateaued (Figure 3a). The flavonoid yield peaked at a spraying ratio of 1:1.75 (g/g), which was significantly higher than that of all other groups (*p* < 0.05). This pattern can be explained by the progressively enhanced adsorption of flavonoids from the ILLE concentrate onto the WT matrix as the spraying ratio increased. However, once the spraying ratio exceeded the optimum, the WT substrate approached adsorption saturation, and excess concentrate could not be effectively loaded. Consequently, the total flavonoid yield no longer increased and stabilized.

#### 3.3.3. Effect of Spraying Temperature on Flavonoid Yield in IT

With increasing spraying temperature, the flavonoid yield in IT exhibited an initial increase followed by a plateau (Figure 3b). The flavonoid yield reached its maximum at 50 °C, and further temperature elevation did not result in a statistically significant change (*p* > 0.05). This trend can be explained by the adsorption characteristics of flavonoids, which is often an endothermic process; thus, moderate heating can facilitate adsorption. However, above 50 °C, flavonoids may become partially ionized and/or exhibit increased molecular mobility, which can reduce their affinity for the tea surface and promote desorption. As a consequence, the net adsorption of flavonoids no longer increases. This trend is consistent with that reported by Beeler et al. in their study on the adsorption of cocoa flavonoids [19].

#### 3.3.4. Effect of Drying Temperature on Flavonoid Yield in IT

As the drying temperature increased, the flavonoid content in IT exhibited an inverted U-shaped trend, increasing initially and then decreasing (Figure 3c). The maximum flavonoid content was achieved at 105 °C and was significantly higher than that obtained at other drying temperatures (*p* < 0.05). This pattern can be attributed to enhanced molecular thermal motion at elevated temperatures, which facilitates diffusion-driven mass transfer of flavonoids from ILLE into the WT matrix. However, when the temperature exceeded 105 °C, heat-induced degradation became apparent, leading to oxidative deterioration of flavonoid compounds [20] and, consequently, a reduction in flavonoid yield.

### 3.4. Sensory Evaluation

As shown in Table 5, IT received higher panel scores than WT across the evaluated sensory attributes under the present trained-panel assessment scheme. In terms of appearance, IT scored 89.3, while WT scored 88.9, indicating comparable performance. Specifically, the infusion of IT exhibited a bright, deep yellow color, whereas WT produced a lighter-colored liquor. This difference may be associated with the enrichment of chlorophyll, flavonoids, and other pigment-related compounds in IT, which collectively intensified the yellow coloration of the tea infusion, consistent with the findings of Wu et al. [21]. Regarding aroma, IT displayed a delicate ILLE aroma accompanied by a rich tea fragrance, with a sensory score of 97.5, higher than the 87.9 of WT. In terms of taste, IT presented a clear, mellow, refreshing, well-layered, and harmonious mouthfeel, with a score of 93.2, higher than the 85.3 of WT. Overall, IT showed higher overall sensory scores than WT in this expert comparative evaluation. These results suggest that incorporation of ILLE may improve the sensory characteristics of WT under the present evaluation conditions.

### 3.5. Biochemical Component Analysis

**Figure 4:**
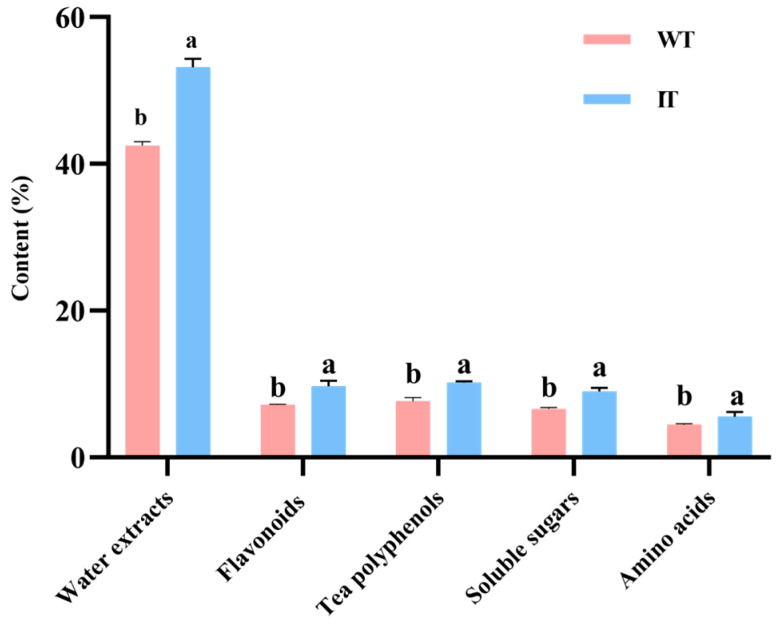
Comparison of the contents of major biochemical constituents in IT and WT. Significant differences at *p* < 0.05 between groups for the same constituent are denoted by different lowercase letters (Student’s *t*-test).

Water extracts, flavonoids, amino acids, tea polyphenols, and soluble sugars are key chemical determinants of tea quality [22]. Among these constituents, water extracts are primary indicators of the total soluble solids content and directly influence the concentration, body, and taste intensity of the infusion [11]. Flavonoids contribute to liquor color, with higher levels generally associated with a brighter yellow appearance [21]. Amino acids, as major flavor-active compounds, primarily impart umami and freshness [23], whereas soluble sugars help mitigate bitterness and astringency by counterbalancing the effects of polyphenols, thereby enhancing mellow and sweet taste attributes [24]. In contrast, tea polyphenols are important contributors to bitterness and astringency [24]. As shown in Figure 4, compared with WT, IT exhibited significantly higher contents of water extracts, amino acids, flavonoids, tea polyphenols, and soluble sugars, with increases of 25.2%, 25.6%, 35.7%, 32.9%, and 18.3%, respectively (*p* < 0.05). These results indicate that during the spray-drying process, WT adsorbed multiple quality-related constituents from ILLE, leading to compositional enrichment. Although tea polyphenols increased in IT, the overall taste of tea depends on the balance among amino acids, soluble sugars, polyphenols, and aroma-active compounds. Therefore, the higher polyphenol content in IT does not necessarily imply poorer palatability when other taste-balancing compounds are also enriched. This compositional trend is consistent with the sensory observations presented above (Table 5).

### 3.6. Analysis of Non-Volatile Compounds

#### 3.6.1. Non-Targeted Profiling of Non-Volatile Metabolites in IT and WT

**Figure 5:**
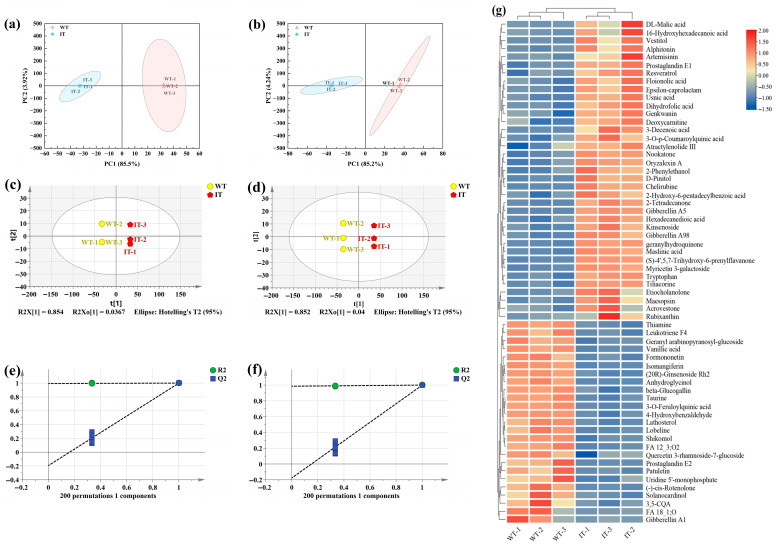
Analysis of non-volatile metabolites in the two sample groups: (**a**,**b**) PCA score plots in positive and negative ion modes; (**c**,**d**) OPLS-DA score plots in positive and negative ion modes; (**e**,**f**) OPLS-DA permutation plots in positive and negative ion modes; (**g**) clustered heatmap of 62 differential metabolites.

In this study, we conducted LC–MS-based, non-targeted metabolomics profiling to assess variations in the non-volatile metabolite profiles of IT and WT. A total of 3230 metabolite features were identified, including 1482 detected in positive ion mode and 1748 in negative ion mode. Among these, 188 metabolites were confidently annotated at the MS/MS level. To further investigate metabolic differences between the two tea samples, multivariate statistical analyses were performed on the complete non-volatile metabolite dataset. PCA score plots demonstrated distinct separation between IT and WT in both positive and negative ion modes (Figure 5a,b), revealing notable variations in their non-volatile metabolite compositions. Orthogonal partial least squares discriminant analysis (OPLS-DA), a supervised modeling technique, further improved group differentiation. Corresponding score plots also highlighted pronounced differences in non-volatile constituents between IT and WT (Figure 5c,d), consistent with the PCA observations. Additionally, cross-validation confirmed that the OPLS-DA models were robust and reliable (Figure 5e,f), supporting the subsequent identification of differential metabolites based on VIP scores.

#### 3.6.2. Identification and Characterization of Differential Metabolites

To systematically compare the non-volatile metabolite profiles of IT and WT, differential metabolites were screened using the following criteria: variable importance in projection (VIP) > 1, *p* < 0.05, and fold change (FC) ≥ 2 or ≤0.5. Based on these thresholds, 62 different non-volatile metabolites were identified. Hierarchical clustering analysis generated a heatmap (Figure 5g), revealing that 37 metabolites were upregulated and 25 downregulated. These metabolites were further classified into chemical categories, including 16 terpenoids, 11 organic acids, 10 flavonoids, 4 amino acids and derivatives, 5 alkaloids, 4 esters, 2 nucleotides, and 10 other compounds.

#### 3.6.3. Association Between Differential Metabolites and Sensory Quality

Non-volatile compounds form the material basis for the rich and full mouthfeel of tea infusions. Among them, organic acids, flavonoids, alkaloids, amino acids, and phenolic compounds are widely recognized as key taste-active constituents due to their direct contributions to critical sensory attributes, including taste intensity, freshness/umami, bitterness/astringency, and aftertaste [25]. Notably, several non-volatile metabolites closely linked to sensory quality were markedly enriched in IT (Table S1). Organic acids (e.g., DL-malic acid and 3-decenoic acid), along with specific flavonoids and amino acids, were significantly elevated, collectively supporting the freshness, astringency, and lingering sweet aftertaste of the infusion. In particular, DL-malic acid increased by 5.3-fold, which may enhance proton dissociation and stimulate oral sour-taste receptors, thereby strengthening the refreshing taste perception [23]. Myricetin 3-galactoside was upregulated by 3.69-fold and has been reported to modulate both bitterness and umami [26]. (S)-4′,5,7-Trihydroxy-6-prenylflavanone increased by 5.98-fold; as a flavonoid, it can contribute to liquor color development and promote a brighter yellow appearance [21]. Tryptophan increased approximately 3-fold, potentially enhancing sweet–umami notes [23], while 2-phenylethanol increased by 2.24-fold, which may impart a pronounced floral aroma and a smoother mouthfeel [27]. In contrast, several phenolic acids commonly associated with bitterness and astringency (e.g., 3,5-Dicaffeoylquinic acid and vanillic acid) were decreased, potentially contributing to improved palatability [28]. Collectively, these metabolite-level shifts help account for the observed increases in water extractables, amino acids, and flavonoids in IT, consistent with its enhanced sensory performance.

Notably, IT exhibited enrichment of several secondary metabolites that have been previously reported in the literature to possess bioactive properties, including usnic acid [29], D-pinitol [30], chelirubine [31], artemisinin [32], resveratrol [33], atractylenolide III [34,35], gibberellin A98 [36], and genkwanin [37]. Their relative abundances in IT were 5.08-, 3.03-, 2.93-, 2.64-, 2.41-, 2.10-, 2.09-, and 2.05-fold higher than those in WT, respectively. Previous studies have associated these compounds with antioxidant, anti-inflammatory, antimicrobial, metabolic regulatory, or other bioactivity-related properties. However, it should be noted that the present study did not directly evaluate these biological effects in IT. Therefore, these enriched metabolites should be interpreted as compounds with potential functional relevance, rather than as direct evidence of confirmed pharmacological effects of the product itself.

During processing, the incorporation of ILLE into IT promoted the enrichment of multiple secondary metabolites reported to have potential bioactive relevance. These findings suggest that IT may possess enhanced compositional characteristics compared with WT and provide a chemical basis for future investigations into its possible functional properties. Further biological validation will be required to confirm whether these enriched metabolites translate into measurable health-related effects.

### 3.7. Analysis of Volatile Compounds

#### 3.7.1. Volatile Metabolite Profiling of IT and WT

**Figure 6:**
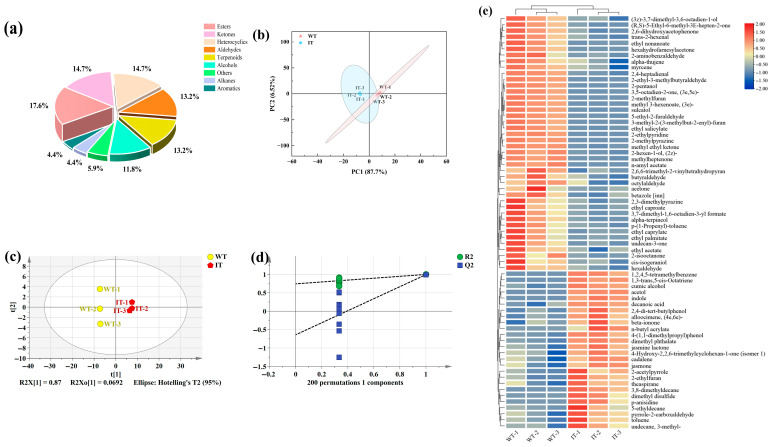
Analysis of volatile metabolites in the two sample groups: (**a**) classified distribution chart; (**b**) PCA score plot; (**c**) OPLS-DA score plot; (**d**) OPLS-DA permutation plot; (**e**) clustered heatmap of 68 different metabolites.

The volatile flavor compounds in IT and WT were characterized using GC–MS. A total of 68 volatile compounds were identified, comprising 12 esters (17.6%), 10 ketones (14.7%), 10 heterocyclic compounds (14.7%), 9 aldehydes (13.2%), 9 terpenes (13.2%), 8 alcohols (11.8%), 3 alkanes (4.4%), 3 aromatic hydrocarbons (4.4%), and 4 compounds of other classes (5.9%) (Figure 6a).

PCA was conducted on all detected volatile compounds from the two samples. The first and second principal components accounted for 40.9% and 19.5% of the total variance, respectively (Figure 6b). The IT and WT samples were clearly separated in the score plot, indicating that ILLE adsorption treatment markedly altered the volatile metabolite profile of WT. Further analysis using orthogonal partial least squares discriminant analysis (OPLS-DA) yielded an even clearer classification: all samples fell within the 95% confidence ellipse, with IT and WT forming distinct clusters on opposite sides, demonstrating significant differences in their volatile metabolite composition (Figure 6c). Cross-validation of the model gave a Q^2^ intercept < 0, confirming its stability and reliability without overfitting (Figure 6d).

The 68 identified volatile metabolites were standardized and visualized in a clustered heatmap (Figure 6e) showing their relative abundance in IT and WT. The results distinctly separated IT and WT into two groups. Compared with WT, 26 volatile metabolites were significantly upregulated in IT (Table S2): five terpenes, four alcohols, three heterocyclic compounds, three esters, three alkanes, two aromatic hydrocarbons, one aldehyde, one organic acid, one ketone, and three compounds of other classes. Among these, terpenes and alcohols predominated, followed by heterocyclic compounds, esters, and alkanes. This distribution pattern aligns with previous studies on Osmanthus black tea and pomelo flower green tea, which identified terpenes, alcohols, and heterocyclic compounds as key characteristic volatiles [38,39].

Previous studies have indicated that volatile terpenes are key contributors to the sweet and floral aroma of tea and play a critical role in enhancing its quality [40]. Alcohols generally form the primary basis for refreshing notes [27], and their increased content in IT is mainly attributed to the adsorption and retention of naturally occurring alcohols from ILLE during processing. Tea leaves, as highly porous solid adsorbents with extensive microporous networks, exhibit enhanced adsorption capacity [41,42], facilitating the enrichment of terpenes, heterocyclic compounds, esters, and alkanes in IT. Notably, heterocyclic compounds often impart nutty, caramel, and sweet aroma characteristics [43], whereas esters are major contributors to floral and fruity notes [27]. Collectively, the spraying and adsorption of ILLE introduce multiple aroma-active components into the WT matrix, enabling their effective loading and synergistic retention, thereby driving a more complex and layered flavor profile.

#### 3.7.2. Analysis of Differential Volatile Metabolites

The introduction of ILLE resulted in a significant increase in the relative abundance of 26 volatile compounds in IT (Table S2), with the most prominent enrichment observed for 1,3-trans,5-cis-octatriene and 1,2,4,5-tetramethylbenzene—312-fold and 295-fold higher than in WT, respectively. These compounds are rarely detected in white tea and are presumed to originate primarily from the exogenous addition of ILLE. Additionally, several endogenous tea aroma compounds, including β-ionone, jasmonolide, jasmonone, dimethyl disulfide, toluene, and indole, were significantly upregulated in IT. Among these, β-ionone, jasmonolide, and jasmonone contribute characteristic floral and fruity notes; dimethyl disulfide imparts fruity and fresh aromas; and toluene exhibits a sweet and mellow note. Notably, indole, a key aroma component in jasmine and Osmanthus teas [44], exhibits sweet and floral characteristics at low concentrations but can develop an unpleasant fecal odor at higher levels [39,45]. In IT, indole showed a fold change of 3.91, suggesting its relative abundance remained sufficiently low to enhance aroma formation—a trend similarly observed in grapefruit green tea [39].

In summary, ILLE may enhance the aroma quality of IT through a dual mechanism. First, a direct contribution arises from the abundant active compounds and aroma precursors in ILLE, including terpenes, alcohols, and esters. These are physically enriched by the porous WT matrix during spraying and adsorption, subsequently penetrating the internal tissues. Second, an indirect transformation occurs during the subsequent drying process, where moderate heat facilitates Maillard reactions, redox reactions, and isomerization between endogenous WT compounds and exogenous ILLE components [46]. These reactions collectively promote the formation of new aroma compounds and restructure the existing aroma profile.

## 4. Conclusions

This study developed a processing strategy for integrating *Indocalamus latifolius* leaf extract (ILLE) into white tea (WT) to produce a novel *Indocalamus latifolius* leaf–white tea (IT) with distinctive sensory and compositional characteristics. By combining process optimization, sensory evaluation, biochemical analysis, and untargeted metabolomics, the study demonstrated that the incorporation of ILLE can effectively improve the aroma profile, taste quality, and chemical composition of WT. Compared with WT, IT showed a more characteristic and persistent leaf aroma, a smoother and more layered taste, and higher levels of several quality-related constituents. Metabolomic analysis further revealed clear compositional differentiation between IT and WT, including enrichment of multiple metabolites associated with aroma and potential functional relevance. The novelty of this work lies in the development of a spray-assisted enrichment strategy that differs from traditional scenting processes by introducing not only aroma-active compounds but also a broader range of non-volatile metabolites into the white tea matrix. From a practical perspective, this approach provides a new pathway for white tea product innovation and offers a promising strategy for the high-value utilization of *Indocalamus latifolius* leaf resources. Overall, the findings provide a useful basis for process standardization and future development of this novel specialty tea.

## Figures and Tables

**Table 1:** Factors and levels used in the Box–Behnken experimental design.

Level	Factor		
	A: Temperature (°C)	B: Time (min)	C: Liquid-to-Solid Ratio (mL/g)
−1	80	60	45:1
0	90	90	60:1
1	100	120	75:1

**Table 2:** Experimental design and results for response surface analysis.

Test Group	A	B	C	Total Flavonoid Yield (mg/g)
1	0	0	0	4.042
2	0	1	1	3.980
3	0	−1	−1	3.254
4	0	0	0	4.092
5	0	0	0	4.502
6	1	−1	0	3.870
7	0	1	−1	4.476
8	−1	0	−1	2.470
9	0	0	0	4.246
10	0	−1	1	3.224
11	−1	1	0	2.690
12	1	0	1	3.926
13	1	1	0	4.702
14	−1	−1	0	1.756
15	0	0	0	4.558
16	−1	0	1	2.846
17	1	0	−1	3.294

**Table 3:** Analysis of variance for the quadratic polynomial model.

Source of Variance	Sum of Squares	Degree of Freedom	Mean Square	F-Value	*p*-Value	Significance
Model	10.15	9	1.13	6.95	0.0091	**
A	4.55	1	4.55	28.00	0.0011	**
B	1.75	1	1.75	10.79	0.0134	*
C	0.0290	1	0.0290	0.1789	0.6850	
AB	0.0026	1	0.0026	0.0160	0.9028	
AC	0.0164	1	0.0164	0.1009	0.7600	
BC	0.0543	1	0.0543	0.3344	0.5812	
A^2^	2.81	1	2.81	17.29	0.0043	**
B^2^	0.1983	1	0.1983	1.22	0.3065	
C^2^	0.4796	1	0.4796	2.95	0.1293	
Residual	1.14	7	0.1623			
Lack of fit	0.9169	3	0.3056	5.57	0.0652	Not significant
Pure error	0.2194	4	0.0548			
Total	11.29	16				

Note: *p* < 0.05 indicates a significant difference, marked with *; *p* < 0.01 indicates the most significant difference, marked with **. R^2^ = 0.8993; Adj.R^2^ = 0.7699; Adeq precision = 7.9074; Std.Dev. = 0.4029; Mean = 3.64; C.V.% = 11.06.

**Table 4:** Influence of spraying ratio on the adsorption performance of raw white tea.

Spraying Ratio (g/g)	Adsorption Capacity (g)	Adsorption Rate (%)	Time to Adsorption Saturation (min)
1:0.25	0.99 ± 0.2 ^g^	19.8 ± 4 ^f^	6
1:0.5	1.87 ± 0.2 ^f^	37.4 ± 4 ^e^	8
1:0.75	2.85 ± 0.1 ^e^	57 ± 2 ^d^	15
1:1	3.92 ± 0.3 ^d^	78.4 ± 6 ^c^	20
1:1.25	4.02 ± 0.2 ^c^	80.4 ± 4 ^c^	25
1:1.5	4.83 ± 0.1 ^b^	96.6 ± 2 ^b^	25
1:1.75	5.29 ± 0.1 ^a^	105.8 ± 2 ^a^	25
1:2	5.31 ± 0.2 ^a^	106.2 ± 4 ^a^	30

Note: Different lowercase letters in the same column indicate significant differences (*p* < 0.05).

**Table 5:** Sensory evaluation results for IT and WT.

Type	Tea Leaves	Tea Infusion	Appearance		Aroma		Taste	
			Comments	Score	Comments	Score	Comments	Score
IT	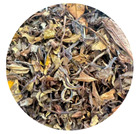	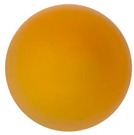	Bright yellow infusion	89.3	delicate ILLE aroma accompanied by rich tea fragrance	97.5	clear, mellow, and refreshing; well-layered and harmonious	93.2
WT	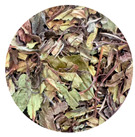	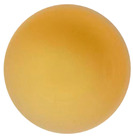	pale yellow infusion	88.9	rich tea aroma	87.9	clear and relatively strong taste	85.3

## Data Availability

The original contributions presented in the study are included in the article/Supplementary Materials; further inquiries can be directed to the corresponding authors.

## Supplementary Materials

The following supporting information can be downloaded at: https://www.mdpi.com/article/10.3390/foods15101676/s1, Figure S1: Calibration curves for biochemical assays: (a) Gallic acid standard curve; (b) L-theanine standard curve; (c) Glucose standard curve; (d) Rutin standard curve; Figure S2: Scatter plot of data points from the response surface analysis; Table S1: Information on 62 differential non-volatile metabolites; Table S2: Information on 68 differential volatile metabolites.
