# Supplementary material for: Process Optimization and Quality Characterization of Indocalamus latifolius Leaf–White Tea
Source: Foods. 2026 May 11;15(10):1676. doi: 10.3390/foods15101676 (PMC13205205; doi:10.3390/foods15101676)
Supplement: Supplementary file 1 [file foods-15-01676-s001.zip › foods-4230532-supplementary/Supplementary Figures.pdf]

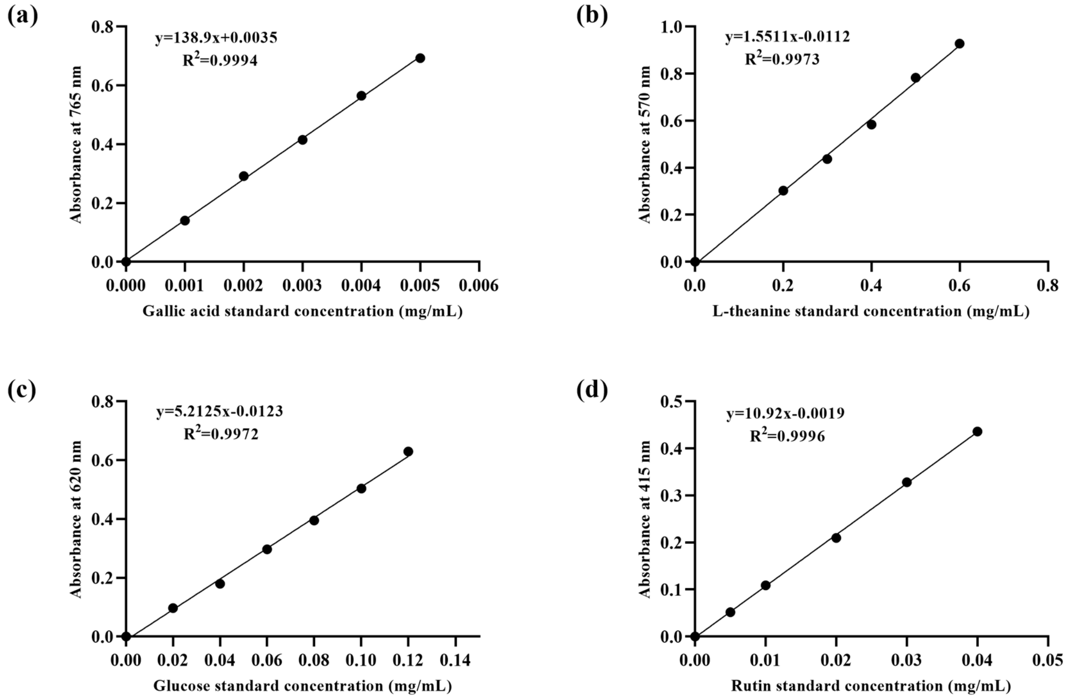

Figure S1. Calibration curves for biochemical assays. (a) Gallic acid standard curve for total polyphenols determination (Folin–phenol method, absorbance at 765 nm); (b) L-theanine standard curve for amino acids determination (ninhydrin method, absorbance at 570 nm); (c) Glucose standard curve for soluble sugars determination (anthrone-sulfuric acid method, absorbance at 620 nm); (d) Rutin standard curve for total flavonoids determination (aluminum trichloride method, absorbance at 415 nm).

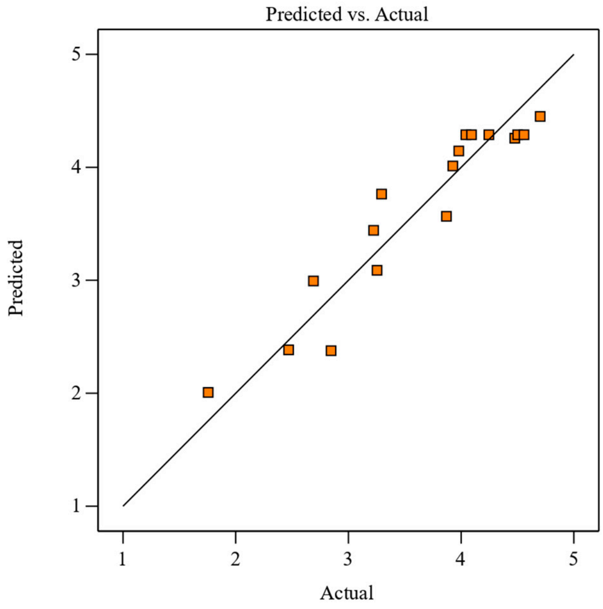

Figure S2. Scatter plot of data points from the response surface analysis.
